# A Letter to Subscribers

**Published:** 1914-12

**Authors:** F. E. Daniel


### A Letter to Subscribers.

Dear Friends and Patrons: For a long while I have been wishing that you might enjoy the benefit of my exchanges.

You know that the ensign of the Texas Medical Journal is Helpfulness, Originality and Progress. I believe I have found a plan by which these three things can be realized every day in the month and every month in the year for every reader of the "Red Back" who wishes to take advantage of the opportunity.

For some time past I have been elated over the prospect of being of real service to you aside from the service of giving you a clean progressive and helpful journal each month.

It is my plan to establish for you what is known as the Package Library System, and thus enable you to enjoy the benefit of the hundreds of medical journals which I receive each month from the various sections of the United States, Canada and Europe. These journals contain articles on various subjects, from the pens of the best men in the profession, men who are putting theories into action. Of course no one physician can take them all, nor could he read them if he should take them, but by the package library system every one can take advantage of the best articles that appear on any subject in which he may be interested.

Let us suppose, for instance, that you are invited to prepare a paper on some of the diseases to which humanity is subject. You would like to be accommodating and you believe it to be the honorable duty of every physician to contribute his share toward making his society interesting. You know that your experience has been rather limited on this particular subject, and that while you can prepare an article, you feel that it will be dull and rather uninteresting. You perhaps have no late books on the subject, and you have no time to study them even if you had them. Or you have been notified that you will be expected to discuss a certain subject at your next society meeting, wouldn't you like to be well armed with facts and figures from the very best authorities? Wouldn't you like to know that by writing for and paying the postage you could have these facts at hand in a concise form in plenty of time to enable you to prepare yourself intelligently?

Suppose that you have a case that is giving you trouble, you have consulted the physicians of your town or surrounding country, you have carefully consulted your books all to no avail, wouldn't you like to be able to sit down in your office and read the thoughts, suggestions and experiences of the best men in the profession on this particular subject? What could be more helpful and what would so enable you to study the different phases of the case, as would some half dozen good articles from men who have been so situated that they have had more experience and have been able to give more of their time to this particular subject than you have done.

If there are only a few men in your society who have the talent or the time to write papers, there are many who would be encouraged if they were asked to select and read some article on the topic you had up for discussion. They could have access to the very best and latest through the package library system.

Each month the exchanges will be gone over and the various articles of interest will be clipped and classified according to subjects, so that when you write that you wish information on any subject I can send you forward immediately a package that will contain new and original stuff, and as most magazine articles are short you will have in concise form all the very latest thought of the best men.

This will enable you to keep abreast of the times. You may not be able to buy many books, and you are so busy that you have time to read only a few, but there is no excuse for your falling behind the advancing profession. You do not have to fall behind; you should be ashamed to admit to yourself that you have fallen behind, for the man who practices medicine in any of its branches should feel honor bound to keep himself informed. If he does not do so, he should be ostracized by the profession. Human life is too dear to be dealt with by any man who is so lazy mentally that he quits reading and studying when he receives his diploma. Such a man should join the army. He isn't fit for anything but machine work, and the sooner his kind is killed off the better for the world in general.

It is my purpose to help you in any line of thought in which you may be interested and to place in your hands on short notice the best and latest theories from the most advanced thinkers.

By this plan the humblest doctor in every village, hamlet and town may be enabled to give his patients the advantage of the latest scientific thought. But—and now please note well what I am going to say—I *must know* that *you will take advantage* of this free package library system before I go to work to establish it.

Of course, if you will stop and think a moment, you will realize that it means an immense amount of work, to say nothing of the expense involved in putting in a filing system and having help to attend to the mailing and recording.

The package library will be free to the "Red Back" readers, except for the postage, which will, of course, be a small amount.

I am quite willing, in fact I am anxious to do this for you, but I must be assured that you are sufficiently interested to write and tell me that you approve of this plan, and that you will be glad to use it in your work; also, that a sufficient number of the doctors are interested to make it worth while for the time and thought I shall give to it. I do not wish to go to the trouble and expense unless it is going to mean a more enlightened and progressive profession.

I am of the opinion that a well planned package library system, such as I contemplate establishing, will materially assist the physicians of Texas in becoming known and recognized as the most progressive and intelligent body of physicians in the United States.

The medical colleges of the State and the State Board of Medical Examiners are doing all in their power to bring about this desired end. They are laying foundations upon which every man may build a noble structure that will be an honor to his Alma Mater and bring distinction to the professional body of his State.

So, if this idea appeals to you as a man or a society of men, please write and let me know that you approve of it and that you mean to take advantage of it. Of course, if you do not feel enough interest to write your approval and assure me of your co-operation in this matter, I shall do nothing more about it. What have you to say about it? Shall we have the package library system or not? It is up to you.

But please bear in mind, dear friends, that I have not telepathic powers, and I shall not know what you think of this proposition unless you write and tell me.

MRS. F. E. DANIEL.

